# Machine Learning–Driven Prognostic Model Integrating Lymphocyte‐to‐C‐Reactive Protein Ratio and TNM Staging in Gallbladder Cancer

**DOI:** 10.1002/cam4.71646

**Published:** 2026-02-20

**Authors:** Mingyang Wang, Zhengyu Chen, Fusheng E, Jun Gu, Runfa Bao

**Affiliations:** ^1^ Department of General Surgery Xinhua Hospital Affiliated to Shanghai Jiao Tong University School of Medicine Shanghai China; ^2^ Shanghai Key Laboratory of Biliary Tract Disease Research Shanghai China; ^3^ Department of General Surgery, Shigatse People's Hospital Shigatse China

**Keywords:** gallbladder cancer, machine learning, preoperative clinical information, preoperative hematological parameters, prognosis, radical cholecystectomy

## Abstract

**Background:**

A comprehensive preoperative assessment of the patient's physical condition is crucial for predicting the prognosis of patients undergoing radical cholecystectomy for gallbladder cancer (GBC). This study aimed to develop a prognostic model integrating preoperative hematological parameters and clinical information to predict postoperative survival in patients with GBC.

**Methods:**

Patients who underwent radical cholecystectomy for GBC between 2000 and 2024 at Xinhua Hospital, affiliated with Shanghai Jiao Tong University School of Medicine, and Shigatse People's Hospital were included in this study. Data on demographic features, clinical parameters, laboratory results, and clinical outcomes were collected. Univariate and multivariate Cox regression analyses, time‐dependent ROC curve analysis, and the least absolute shrinkage and selection operator (LASSO) regression were used to identify the key factors for model development. Various machine learning models were constructed based on these findings. Internal validation assessed model stability, while clinical decision analysis evaluated its practical utility.

**Results:**

A total of 184 patients were included, with a mean age of 67 years. Key predictors identified through univariate and multivariate Cox regression, time‐dependent ROC, and LASSO analyses were the lymphocyte‐to‐C‐reactive protein ratio (LCR) and tumor‐node‐metastasis (TNM) staging. The best‐performing model was logistic regression, with the following area under the curve (AUC) values: for the training set, 0.785 at 1 year, 0.853 at 2 years, and 0.873 at 3 years; and for the test set, 0.800 at 1 year, 0.870 at 2 years, and 0.872 at 3 years. Clinical decision analysis confirmed the model's clinical applicability.

**Conclusion:**

The machine learning model incorporating LCR and TNM staging is a robust tool for predicting postoperative survival following radical resection for GBC.

## 1 Introduction

Gallbladder cancer (GBC) is the most common malignancy of the biliary tract and ranks among the top six gastrointestinal cancers [1]. China, with its high incidence of GBC, accounts for 24.7% of the global cases [2]. The incidence rate of GBC in China is approximately 3.82 per 100,000 individuals [3]. Despite advancements in medical treatment, GBC remains associated with a poor prognosis, primarily due to late‐stage diagnosis, high invasiveness, and limited treatment options. The 5‐year survival rate for GBC typically remains below 10% [4, 5]. Currently, radical cholecystectomy is the most effective treatment for curative resection [6, 7]. However, it is often followed by a poor postoperative prognosis and a relatively short survival duration. Therefore, it is crucial to develop reliable prognostic models using preoperative patient data, thereby significantly enhancing clinical decision‐making and optimizing public health resource allocation.

Numerous studies have demonstrated that both the tumor‐node‐metastasis (TNM) staging system and various hematological parameters are strongly associated with the prognosis of patients undergoing radical resection [8, 9, 10]. However, two key issues remain. First, while the TNM staging system offers valuable anatomical insights into tumor spread and invasion, it does not fully account for the physiological condition of the patient [11]. Second, although hematological parameters provide some insight into the patient's physiological status, they fail to capture the full complexity and heterogeneity of the tumor [12]. Additionally, although several hematological markers have been linked to survival outcomes, not all of these indicators are suitable for accurately predicting postoperative prognosis in patients with GBC. Thus, this study aimed to identify the most significant hematological and clinical parameters to establish an accurate and concise prognostic model for predicting survival following radical cholecystectomy in GBC patients. The development of such models is essential for improving clinical management and optimizing treatment decisions for patients with GBC.

This study had two primary objectives: (1) to identify key prognostic indicators from a broad range of hematological and clinical parameters using univariate and multivariate Cox regression analyses, followed by LASSO regression for variable selection, and (2) to train models using eight different machine learning algorithms and select the best‐performing model by comparing their predictive efficacy.

## 2 Materials and Methods

**FIGURE 1:**
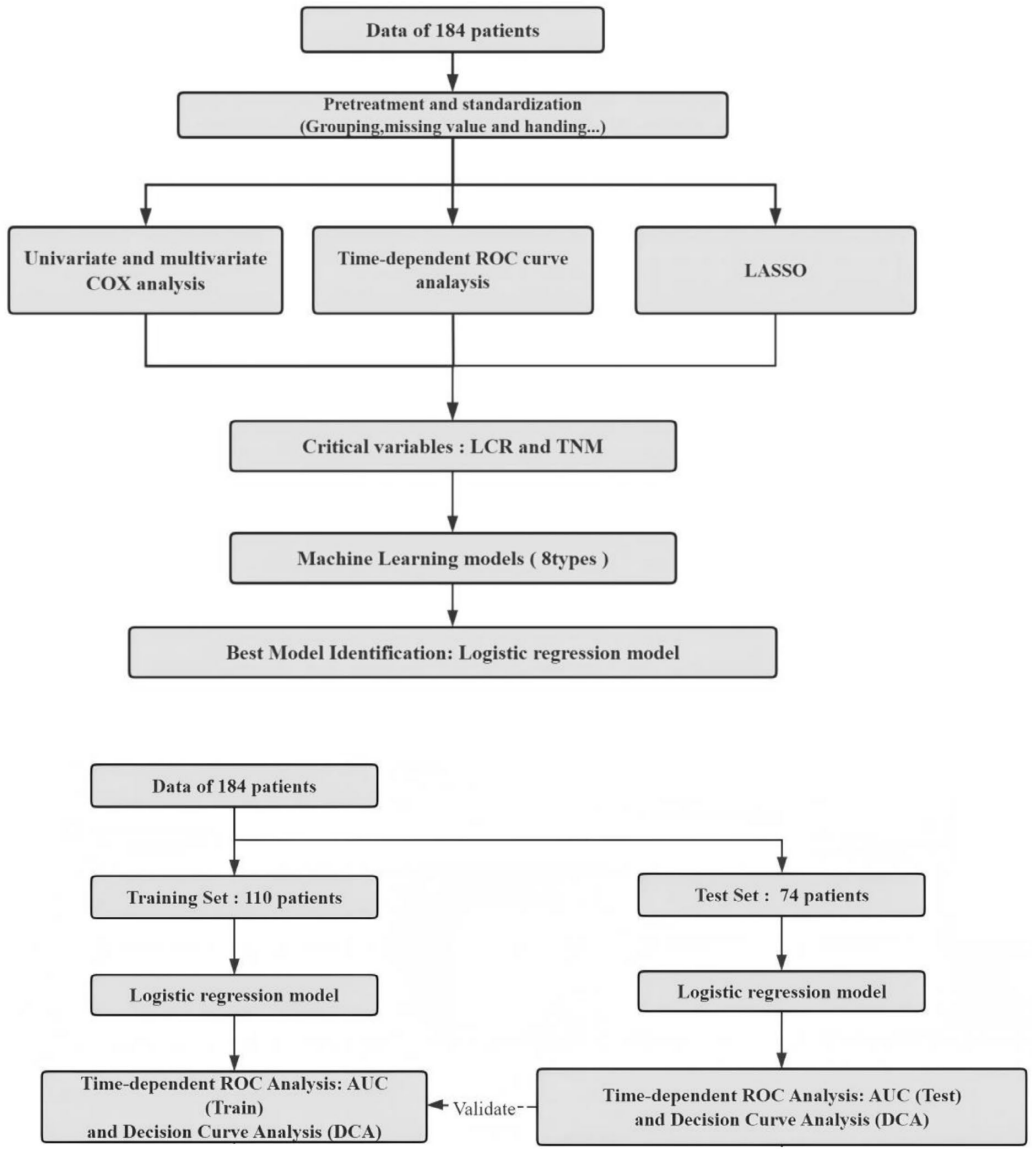
Flowchart of analyisis. LCR: Lymphocyte‐to‐CRP Ratio, LASSO: Least Absolute Shrinkage and Selection Operator.

The overall study design and workflow are illustrated in Figure 1.

### 2.1 Study Population

This study included a cohort of 184 patients who underwent radical cholecystectomy for the treatment of GBC between 2000 and 2024 at Xinhua Hospital, affiliated with Shanghai Jiao Tong University School of Medicine (*n* = 172) and Shigatse People's Hospital (*n* = 12). All patients met the following inclusion criteria: (i) a GBC diagnosis confirmed by pathology, and (ii) no neoadjuvant chemotherapy or radiotherapy administered prior to surgery.

Exclusion criteria were as follows: (1) presence of other malignancies; (2) absence of laboratory data within 1 week before surgery; (3) preoperative infection, including cholecystitis or cholangitis; (4) presence of other hematological disorders; and (5) receipt of palliative resection.

This study was approved by the Ethics Committee of the Institutional Review Board of Xinhua Hospital, affiliated with Shanghai Jiao Tong University School of Medicine (XHEC‐D‐2024‐081). The study was also approved by the Ethics Committee of Shigatse People's Hospital (2023RKZRMYY08M004).

### 2.2 Data Collection and Follow‐Up

Demographic data, comorbidities, vital signs, and laboratory findings were extracted from the Hospital Information Systems (HIS) of both hospitals. Preoperative laboratory values, obtained within 1 week prior to radical cholecystectomy, included measurements of C‐reactive protein (CRP), white blood cells, neutrophils, lymphocytes, platelets, hemoglobin, total bilirubin (TB), and albumin. Based on these laboratory results, new metrics were derived, as shown in Table 1.

**TABLE 1:** Metric abbreviation and calculation method.

Metric abbreviation	Calculation method	Description
LCR (Lymphocyte to CRP Ratio)	Lymphocyte count (number/L)/CRP (mg/L)	The ratio of lymphocyte count to C‐reactive protein level
NLR (Neutrophil to Lymphocyte Ratio)	Neutrophil count (number/L)/Lymphocyte count (number/L)	The ratio of neutrophil count to lymphocyte count
PLR (Platelet to Lymphocyte Ratio)	Platelets count (number/L)/Lymphocyte count (number/L)	The ratio of platelet count to lymphocyte count
LWR (Lymphocyte to White Blood Cell Ratio)	Lymphocyte count (number/L)/White Blood Cell count (number/L)	The ratio of lymphocyte count to white blood cell count
NWR (Neutrophil to White Blood Cell Ratio)	Neutrophil count (number/L)/White Blood Cell count (number/L)	The ratio of neutrophil count to white blood cell count
CAR (CRP to Albumin Ratio)	C‐Reactive Protein (mg/L)/Albumin (g/L)	The ratio of C‐reactive protein to albumin
PNI (Prognostic Nutritional Index)	PNI = 1 × Albumin level (g/L) + 5 × Total Lymphocyte Count (per L)	A combined index based on albumin level and total lymphocyte count
mGPS (modified Glasgow Prognostic Score)	Based on serum markers: Score 0: CRP ≤ 10 mg/L and Albumin ≥ 35 g/L Score 1:CRP > 10 mg/L with normal Albumin Score 2: CRP > 10 mg/L with Albumin < 35 g/L	A prognostic score based on CRP and albumin levels

Patients were followed up through telephone calls, outpatient visits, or inpatient visits until December 2024 or until the patient's death. Follow‐up occurred every 3 months for 3 years post‐surgery.

### 2.3 Predictor Variables

All candidate predictor variables were obtained preoperatively. These included demographic variables (age and sex), tumor‐related factors (TNM stage), routine laboratory parameters (total bilirubin, C‐reactive protein, white blood cell count, neutrophil count, lymphocyte count, platelet count, hemoglobin, and albumin), and derived inflammatory indices (LCR, CAR, NLR, PLR, PNI, and mGPS).

### 2.4 Outcome Variables

The primary outcomes were postoperative survival at 1, 2, and 3 years. For each time point, survival status was defined as a binary outcome (alive or deceased).

### 2.5 Baseline Table Construction

Survival durations for patients who were still alive at the time of follow‐up were treated as censored observations. Statistical analyses were conducted using SPSS version 27. To compare baseline characteristics between groups, the twogrps () function in the R software package GBCgrps was used to allow for automatic and accurate comparison of data. Continuous variables were presented as mean ± standard deviation or median (inter‐quartile range). For comparisons between groups, the independent samples *t*‐test or the Wilcoxon rank‐sum test was used, depending on the distribution of the data. Categorical variables were expressed as frequencies and percentages, with group comparisons performed using the chi‐square test.

### 2.6 COX Regression Analysis

Univariate Cox regression analyses were initially performed for each variable. Significant variables from these analyses were then included in multivariate Cox regression to control for confounding factors and identify independent predictors of survival. The results were presented as hazard ratios (HRs) with 95% confidence intervals (CIs) and *p*‐values.

### 2.7 Data Preprocessing for Time‐Dependent ROC Analysis

For time‐dependent ROC analysis, postoperative survival status at 1, 2, and 3 years was used to construct time‐specific binary outcomes. This stratification allowed for an evaluation of the time‐dependent performance of various clinical characteristics across different patient subsets.

### 2.8 LASSO Regression

All variables were standardized using *z*‐score normalization prior to analysis. LASSO Cox regression with L1 regularization was applied for feature selection, and the optimal penalty parameter (λ) was selected using 10‐fold cross‐validation based on the minimum partial likelihood deviance.

### 2.9 Various Machine Learning Analysis

Eight machine learning models were used for further analysis: logistic regression, naïve Bayes, multilayer perceptron (MLP), random forest, support vector machine with radial basis function kernel (SVM‐RBF), boosted trees, k‐nearest neighbors, and decision tree. These selected features were then input into each model for training. Model performance was assessed using Brier score and the area under the curve (AUC).

For internal validation, model stability was further assessed using 5‐fold cross‐validation and 1000‐bootstrap resampling, which was additionally used to obtain smoothed ROC curves and bootstrapped AUC estimates.

### 2.10 Logistic Regression Model Construction and Nomogram Development

To evaluate model performance, the dataset was randomly split into a training set (60%) and a testing set (40%). A logistic regression model was developed using the training set, and a nomogram was constructed. Its performance was then evaluated on the testing set, with specific evaluation metrics including precision, specificity, and AUC.

Moreover, survival calibration plots were constructed for both the training and testing sets to assess the calibration of the predictive model. A Decision Curve Analysis (DCA) was performed to evaluate the clinical utility of the model. To reduce curve fluctuations caused by the limited sample size, ROC curves were additionally smoothed using bootstrap resampling (1000 iterations), and bootstrapped AUC values were reported.

To address class imbalance (76% deceased vs. 24% alive), Synthetic Minority Over‐sampling Technique (SMOTE) was applied as a sensitivity analysis, and ROC performance was re‐evaluated under the SMOTE‐balanced dataset to assess robustness.

## 3 Results

### 3.1 Characteristics of Study Patients

The baseline characteristics of the study cohort are summarized in Table 2. A total of 184 patients were included, comprising 64 (34.78%) males and 120 (65.22%) females. The median survival duration was 18 months, with an interquartile range (Q1, Q3) of 9 to 32 months. Tumors were staged as follows: Stage I, 14.13%; Stage II, 17.39%; Stage III, 47.28%; and Stage IV, 21.20%. At the end of the follow‐up period, 140 (76.09%) patients had died, while 44 (23.91%) patients were still alive.

**TABLE 2:** The characteristics of patients with GBC.

Variables	*N* (%)	Percentage	Variables	Value	Unit
Sex			Age, Median (Q1, Q3)	67 (59, 74)	Years
Male	64	34.78%	Survival Time, Median (Q1, Q3)	18 (9, 32)	Months
Female	120	65.22%	TB, Median (Q1, Q3)	12 (7.75, 18.45)	μmol/L
Status, *n* (%)			CRP, Median (Q1, Q3)	9 (4, 20.5)	mg/L
Survived	44	23.91%	Albumin, Median (Q1, Q3)	38.65 (35.5, 41.6)	g/L
Deceased	140	76.09%	Hemoglobin, Median (Q1, Q3)	124 (117, 135)	g/L
TNM, *n* (%)			Plt, Median (Q1, Q3)	210.5 173.75, 265.5	× 10^9^/L
1	26	14.13%	WBC, Median (Q1, Q3)	6.08 (4.94, 7.3)	× 10^9^/L
2	32	17.39%	Neutrophil, Median (Q1, Q3)	3.9 (2.9, 5.02)	× 10^9^/L
3	87	47.28%	Lymphocyte, Median (Q1, Q3)	1.48 (1.2, 1.9)	× 10^9^/L
4	39	21.20%	NLR, Median (Q1, Q3)	2.5 (1.79, 3.62)	—
*T*, *n* (%)			PLR, Median (Q1, Q3)	138.49 (106.76, 187.77)	—
1	26	14.13%	LWR, Mean ± SD	0.26 (0.19, 0.32)	—
2	44	23.91%	NWR, Mean ± SD	0.64 ± 0.1	—
3	79	42.93%	LCR, Median (Q1, Q3)	0.17 (0.07, 0.45)	—
4	35	19.02%	CAR, Median (Q1, Q3)	0.23 (0.1, 0.57)	—
*N*, *n* (%)			PNI, Mean ± SD	46.42 ± 6.56	—
0	108	58.70%			
1	68	36.96%			
2	8	4.35%			
mGPS, *n* (%)					
0	102	55.43%			
1	60	32.61%			
2	22	11.96%			

### 3.2 Feature Selection and Prognostic Factor Identification Using Cox and LASSO Regression

**FIGURE 2:**
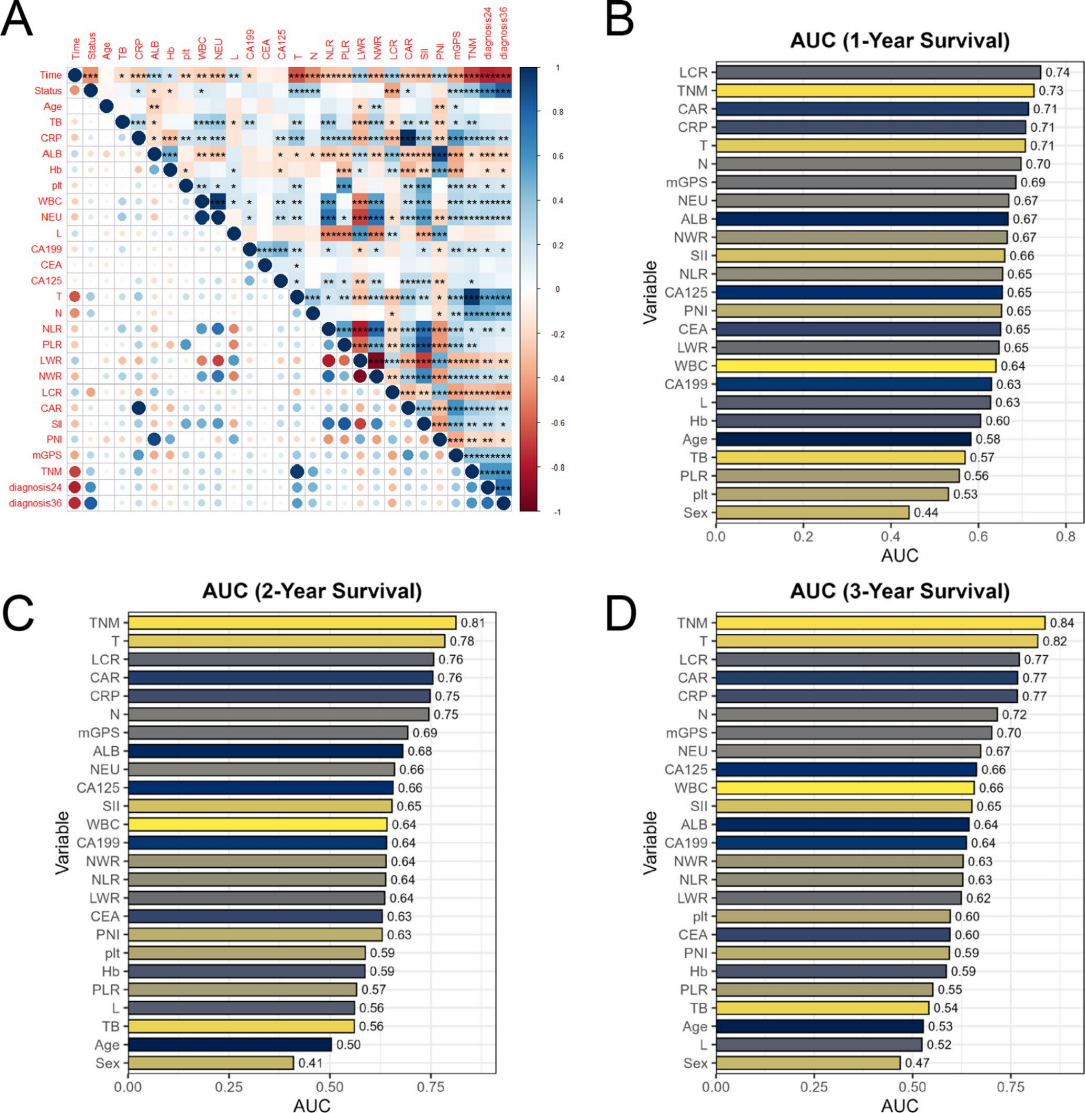
Correlation analysis and time‐dependent ROC performance of clinical and hematological variables in predicting 1‐, 2‐, and 3‐year survival in patients with gallbladder cancer (*n* = 184). (A) Correlation matrix of all variables. Correlation coefficients were calculated using Spearman's method, and significance levels are indicated as follows: *p* < 0.05 (*), *p* < 0.01 (**), *p* < 0.001 (***). (B–D) Time‐dependent ROC analysis for predicting 1‐year, 2‐year, and 3‐year survival, respectively. AUC values represent point estimates derived from logistic regression–based classification models. No hypothesis testing was performed for AUC comparison.

Univariate COX regression analysis followed by multivariate COX regression, incorporating clinically significant variables, identified lymphocyte‐to‐C‐reactive protein ratio (LCR) and TNM staging as independent prognostic factors for survival in patients with GBC following radical cholecystectomy (Table 3, Figure S1). Correlation analysis revealed a moderate‐to‐strong association between LCR and TNM (Figure 2A). Further variable selection using LASSO regression identified both LCR and TNM staging as prognostically relevant variables (Table 4).

**TABLE 3:** Results of univariate COX analysis and multivariate COX analysis.

Characteristic	*N*	Univariate COX analysis		Multivariate COX analysis	
		Hazard ratio (95% CI)	*p*	Hazard ratio (95% CI)	*p*
Age	184	0.998 (0.983–1.014)	0.835		
TB	184	1.003 (1.001–1.006)	0.008	0.997 [0.995, 1.000]	0.055
CRP	184	1.012 (1.008–1.017)	< 0.001	1.023 [0.980, 1.067]	0.302
Albumin	184	0.958 (0.927–0.990)	0.011	0.883 [0.623, 1.252]	0.485
Hemoglobin	184	0.986 (0.976–0.996)	0.005	0.988 [0.974, 1.003]	0.113
plt	184	1.002 (1.000–1.004)	0.058		
WBC	184	1.075 (1.018–1.134)	0.009	0.399 [0.101, 1.584]	0.192
NC	184	1.090 (1.034–1.148)	0.001	3.456 [0.738, 16.183]	0.115
Lymphocyte	184	0.735 (0.533–1.015)	0.061		
NLR	184	1.100 (1.049–1.153)	< 0.001	0.955 [0.809, 1.127]	0.586
PLR	184	1.003 (1.001–1.004)	0.004	1.001 [0.998, 1.005]	0.466
LWR	184	0.020 (0.003–0.143)	< 0.001	4.858 [0.000, 3666760.833]	0.819
NWR	184	33.271 (5.852–189.176)	< 0.001	0.143 [0.000, 43787.245]	0.763
LCR	184	0.411 (0.262–0.643)	< 0.001	0.658 [0.459, 0.943]	0.023
CAR	184	1.477 (1.265–1.726)	< 0.001	0.381 [0.085, 1.707]	0.207
PNI	184	0.996 (0.992–0.999)	0.011	1.104 [0.779, 1.567]	0.578
Sex					
Male	64	1			
Female	120	0.957 (0.665–1.378)	0.814		
TMN					
1	26	1		1	
2	32	1.252 (0.636–2.466)	0.515	1.784 [0.778, 4.090]	0.172
3	87	3.497 (1.995–6.129)	< 0.001	8.111 [3.748, 17.555]	< 0.001
4	39	8.448 (4.338–16.454)	< 0.001	18.096 [7.653, 42.789]	< 0.001
*T*					
1	26	1			
2	44	1.498 (0.822–2.732)	0.187		
3	79	3.118 (1.812–5.368)	< 0.001		
4	35	8.305 (4.332–15.919)	< 0.001		
*N*					
0	108	1			
1	68	3.153 (2.178–4.563)	< 0.001		
2	8	2.942 (1.178–7.347)	0.021		
mGPS					
0	102	1		1	
1	60	2.251 (1.564–3.240)	< 0.001	1.482 [0.903, 2.432]	0.12
2	22	2.464 (1.373–4.423)	0.003	1.149 [0.569, 2.318]	0.699

**TABLE 4:** Variables selected by LASSO regression at different survival endpoints.

Survival endpoint	Selected variables	Coefficient
12‐month	TNM	0.7472
	LCR	−0.3283
	NLR	0.0875
	NEU	0.0442
	Hb	−0.0185
	CRP	−0.0020
	SII	0.00019
	TB	−0.0002
24‐month	TNM	0.6343
36‐month	TNM	1.053
	LCR	−0.2493

### 3.3 Time‐Dependent ROC Curve Analysis

For each of the three time points (1 year, 2 years, and 3 years), survival status was categorized into binary outcomes (survived or deceased). Time‐dependent ROC curve analysis was conducted to evaluate the prognostic value of various clinical and biochemical markers at each time point (Figure 2B–D).

The analysis revealed several key findings. TNM staging showed AUC values of 0.73, 0.81, and 0.84 for the 1‐year, 2‐year, and 3‐year time points, respectively, demonstrating strong discriminatory power at all evaluated time points. LCR showed AUC values of 0.74, 0.76, and 0.77 at 1 year, 2 years, and 3 years, respectively, indicating strong predictive ability across all time periods. Additionally, biochemical markers such as CRP to albumin ratio (CAR), CRP, and modified Glasgow prognostic score (mGPS) demonstrated good prognostic value.

### 3.4 Logistic Regression Model Construction

**FIGURE 3:**
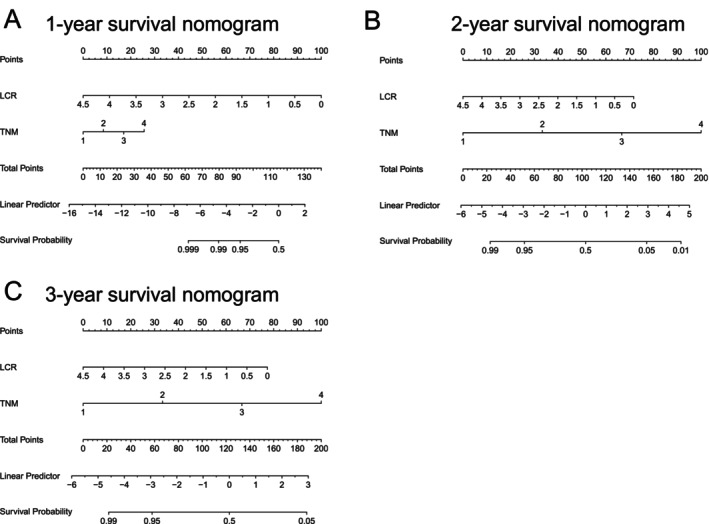
Survival nomograms integrating LCR and TNM stage for predicting postoperative survival in gallbladder cancer.(training set, *n* = 110). Model coefficients and performance metrics are provided in Tables 5 and 6. (A) 1‐year survival nomogram. (B) 2‐year survival nomogram. (C) 3‐year survival nomogram.

Given its superior overall performance among the eight machine learning models, logistic regression was selected for further prognostic modeling (Figure S2A–D). Logistic regression models were developed using the training set to predict survival at 1 year, 2 years, and 3 years postoperatively, incorporating both TNM staging and LCR as predictors. For each time point, a nomogram was constructed (Figure 3A–C). In the training set, the model demonstrated strong discriminatory performance with progressively increasing accuracy over time. The AUC values were 0.785 at 1 year, 0.870 at 2 years, and 0.873 at 3 years, indicating improved predictive performance with longer follow‐up periods. These findings were consistently reproduced in the test cohort, supporting the robustness and generalizability of the model (Table 5).

**TABLE 5:** AUC of models.

Group	1‐Year survival AUC	2‐Year survival AUC	3‐Year survival AUC
Train set	0.785	0.853	0.873
Test set	0.800	0.870	0.872

In the logistic regression models for 1‐year, 2‐year, and 3‐year survival, TNM staging consistently showed a negative impact on survival probabilities (Table 6). Specifically, the coefficients were −0.9346 (*p* = 0.0116) for 1‐year survival, −1.7826 (*p* < 0.001) for 2‐year survival, and −1.8996 (*p* < 0.001) for 3‐year survival. These findings indicate that higher TNM staging is associated with a decreased probability of survival, with the negative effect becoming more pronounced over time.

**TABLE 6:** Coefficients and *p* values of LCR and TNM in survival prediction models.

Endpoint	LCR (coef)	LCR (*p*)	TNM (coef)	TNM (*p*)
1‐Year	3.5111	0.0302	−0.9346	0.0116
2‐Year	0.8203	0.0772	−1.7826	< 0.0001
3‐Year	0.6741	0.0457	−1.8996	< 0.0001

On the other hand, the impact of LCR on survival varied across the different time points. The coefficients for LCR were 3.5111 (*p* = 0.0302) for 1‐year survival, 0.8203 (*p* = 0.0772) for 2‐year survival, and 0.6741 (*p* = 0.0457) for 3‐year survival. These results suggest that while higher LCR values generally correlate with improved survival outcomes, the effect of LCR diminishes over the longer postoperative periods.

### 3.5 Model Validation

**FIGURE 4:**
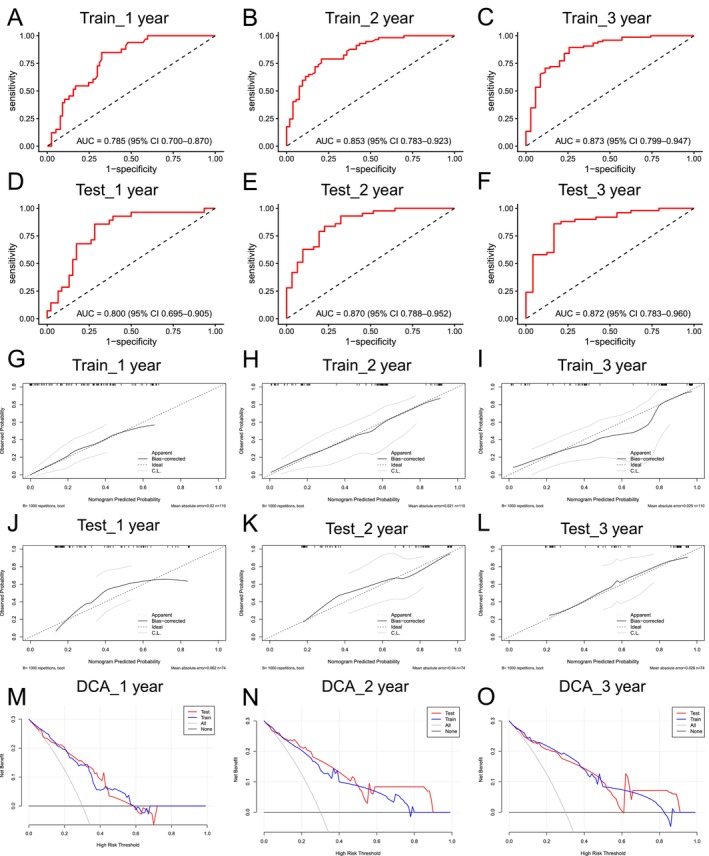
Performance of logistic regression models for survival prediction. (A–F) ROC curves for 1‐, 2‐, and 3‐year survival in training and test sets. (G–L) Calibration plots for 1‐, 2‐, and 3‐year models in training and test sets. (M–s for 1‐, 2‐, and 3‐year outcomes).

The reliability of the models was validated through both ROC curves and calibration plots, based on both the training and testing sets. Both sets demonstrated excellent model performance, with high AUC values indicating strong discriminatory ability (Figure 4A–F). The calibration plots revealed that predicted survival probabilities were well‐aligned with observed outcomes, especially in the bias‐corrected curve, which closely followed the ideal line. This alignment further supports the accuracy and reliability of the predictive models (Figure 4G–L).

DCA for 1‐year, 2‐year, and 3‐year survival (Figure 4M–O) demonstrated a significant net benefit across a range of high‐risk thresholds, indicating that the models perform robustly in both the training and testing datasets.

To assess the influence of class imbalance, SMOTE‐based sensitivity analyses were performed. The SMOTE‐corrected ROC curves yielded AUC values nearly identical to the original model, indicating that imbalance in survival outcomes did not materially affect model performance (Figure S3A–C).

Additionally, bootstrap validation (1000 resamples) demonstrated stable AUC estimates for 12‐, 24‐, and 36‐month predictions, further confirming the robustness of the model (Figure S3D).

### 3.6 Dynamic Changes in Predictor Contributions and Incremental Value of LCR Beyond TNM

**FIGURE 5:**
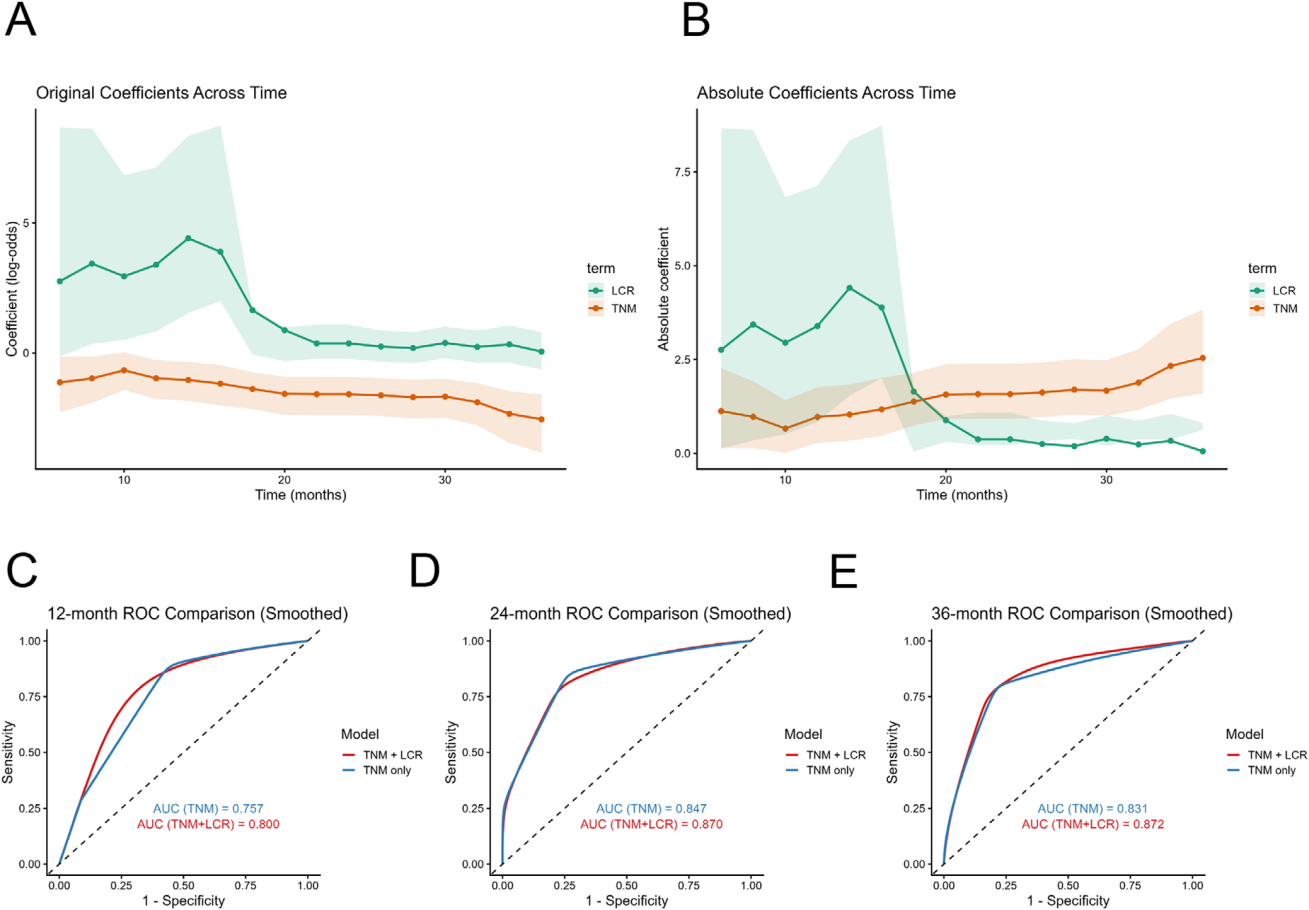
Temporal changes in logistic regression coefficients and external validation of model performance for the TNM+LCR prognostic model. (A) Original regression coefficients of LCR and TNM over time (training set, *n* = 110). (B) Absolute values of regression coefficients (training set, *n* = 110). (C–E) Bootstrap resampling was applied to obtain stable estimates of time‐dependent AUCs for comparing the TNM‐only and TNM+LCR models (test set, *n* = 74).

Analysis of the logistic regression models (Figure 5A,B) showed that the absolute value of the LCR coefficient decreased over time, suggesting that LCR has a greater impact on short‐term prognosis but a diminishing effect on long‐term outcomes. In contrast, the absolute value of the TNM staging coefficient increased, indicating that TNM staging has a relatively smaller effect on short‐term survival but plays a more significant role in long‐term prognosis.

To assess the incremental prognostic contribution of LCR beyond TNM staging, time‐dependent ROC curves were compared using bootstrap‐stabilized AUC estimates (Figure 5C–E). The TNM + LCR model demonstrated consistently higher AUC values than the TNM‐only model at 12, 24, and 36 months. (12‐month: 0.800 vs. 0.757; 24‐month: 0.870 vs. 0.847; 36‐month: 0.872 vs. 0.831). These findings indicate that incorporating LCR provides additional discriminatory benefit beyond TNM staging alone, particularly for short‐term survival prediction.

## 4 Discussion

Tumor, host factors, and treatment play pivotal roles in determining cancer prognosis. While the TNM staging system is widely utilized in clinical practice to assess the prognosis of GBC, it has certain limitations [13]. Recent studies have demonstrated the growing utility of advanced deep learning and hybrid AI frameworks in cancer detection and prognostic modeling across different tumor types, highlighting the expanding role of artificial intelligence in oncologic risk stratification [14, 15]. However, the effectiveness of such AI‐based approaches ultimately depends on the incorporation of biologically relevant and clinically accessible prognostic variables.

Emerging studies have increasingly highlighted the significant role of systemic inflammation, which can be reflected in hematological parameters, in tumorigenesis, tumor progression, and treatment outcomes [16, 17, 18, 19, 20, 21]. Various systemic inflammatory factors have proven to be reliable predictors of cancer prognosis. These factors are often represented through hematological parameters from peripheral blood tests, including neutrophils, lymphocytes, platelets, CRP, albumin, and their combinations [17, 22, 23, 24, 25].

In our study, among the hematological parameters, the LCR emerged as the most critical prognostic indicator. Logistic regression, among the eight machine learning models tested, demonstrated the best performance. The model, incorporating both TNM staging and LCR, achieved an AUC of 0.8. This predictive model holds considerable clinical value in guiding decision‐making for the management of GBC patients and offers valuable insights for future research into prognostic factors.

A growing body of evidence suggests that the LCR ratio serves as a key predictor of tumor prognosis [26, 27, 28, 29]. The link between cancer and inflammation opens several plausible hypotheses regarding the underlying mechanisms. Lymphocytes, as a crucial component of the immune system, are central to combating infections and tumor development. Natural killer (NK) cells play a vital role in cancer immunosurveillance and regulation, utilizing mechanisms to recognize and eliminate tumor cells, particularly those deficient in major histocompatibility complex (MHC) class I molecules [30]. CD4+ helper T cells interact with dendritic cells to shape the tumor microenvironment, regulate immune checkpoint blockade responses, and enhance anti‐tumor effects [31]. The inflammatory response itself contributes to tumor growth and dissemination by promoting angiogenesis, cell proliferation, and inhibiting apoptosis, with CRP serving as a key inflammatory marker that reflects the body's inflammatory state [32, 33]. Numerous studies have shown that elevated levels of CRP correlate with poor prognosis across various cancers, often reflecting more aggressive tumor behavior [34]. As a ratio, LCR amplifies the prognostic signal by simultaneously capturing immune suppression and systemic inflammation, which may explain why it outperforms individual hematologic markers in predicting outcomes in GBC.

Therefore, we propose the combination of preoperative LCR and TNM staging as a new and valuable prognostic tool for predicting the outcomes of perioperative patients with GBC, offering a reliable reference for clinical decision‐making.

This is the first study to combine TNM staging and LCR to construct multiple machine learning models aimed at predicting postoperative survival in GBC patients, ultimately selecting the best‐performing model. Based on both clinical data and advanced AI algorithms, our approach offers a robust and comprehensive methodology. Moreover, TNM staging and LCR are both practical and easily measurable preoperatively, making this model highly applicable in clinical practice. Compared to most models developed in other studies, our model optimized the collinearity between parameters, making it more streamlined and feasible for clinical use. Although the overall cohort size was modest, using only two non‐collinear, biologically meaningful predictors minimizes model complexity and reduces the risk of overfitting—an approach consistent with machine learning recommendations that typically require 10–30 samples per predictor. Thus, the final model (two predictors, 184 patients) achieves an appropriate balance between sample size and model stability while maintaining strong predictive performance [35].

To improve clinical applicability, we developed a simple online risk calculator based on the final logistic regression model using the Shiny framework. By entering only LCR and TNM stage, clinicians can instantly obtain individualized 1‐, 2‐, and 3‐year survival probabilities. The calculator is available at: https://wmy123456.shinyapps.io/GBC_prediction/ and the full source code is provided in the Supporting Informations.

However, several limitations of this study should be acknowledged. First, the sample size was relatively small, which may limit the generalizability of the findings. Nevertheless, gallbladder cancer is a relatively rare malignancy, and only a subset of patients are eligible for radical surgical resection. In this context, the cohort of 184 surgically treated patients included in the present study represents a comparatively substantial sample size. Second, the data were collected from two hospitals, which may introduce potential selection bias. Third, owing to the retrospective design and the lack of predefined data collection protocols, some clinical indicators were missing and therefore excluded from the analysis to ensure model reliability.

To address these limitations, we plan to initiate multicenter, prospective collaborative studies involving additional institutions to further validate and refine the proposed model.

## 5 Conclusion

In conclusion, the combination of preoperative LCR and TNM staging could be an effective prognostic tool for patients with GBC undergoing radical cholecystectomy. This model holds great promise for enhancing clinical decision‐making and guiding the management of GBC patients.

## Author Contributions

**Mingyang Wang:** supervision, conceptualization, methodology, software, investigation, writing – review and editing, writing – original draft. **Zhengyu Chen:** supervision, methodology, software, validation, writing – review and editing, data curation, investigation. **Fusheng E:** formal analysis, supervision, funding acquisition. **Jun Gu:** funding acquisition, visualization, project administration. **Runfa Bao:** conceptualization, funding acquisition, visualization, project administration, resources, writing – review and editing, writing – original draft.

## Funding

This work was supported by Natural Science Foundation of Tibet Autonomous Region, XZ2023ZR‐ZY40 (Z). National Natural Science Foundation of China, 82272691.

## Ethics Statement

This study was approved by the Ethics Committee of Xinhua Hospital, Shanghai Jiao Tong University School of Medicine (Approval No. XHEC‐D‐2024‐081) and the Ethics Committee of Shigatse People's Hospital (Approval No. 2023RKZRMYY08M004).

## Conflicts of Interest

The authors declare no conflicts of interest.

## Supporting information


**Data S1:** Supplementary Figures.

**Data S2:** Supporting Information.

**Data S3:** Supporting Information.

## Data Availability

The data that support the findings of this study are available on request from the corresponding author. The data are not publicly available due to privacy or ethical restrictions.
