# Supplementary material for: Machine Learning–Driven Prognostic Model Integrating Lymphocyte‐to‐C‐Reactive Protein Ratio and TNM Staging in Gallbladder Cancer
Source: Cancer Med. 2026 Feb 20;15(3):e71646. doi: 10.1002/cam4.71646 (PMC12928047; doi:10.1002/cam4.71646)
Supplement: Supplementary file 1 — Data S1: Supplementary Figures. [file CAM4-15-e71646-s001.pptx]

## Slide 1
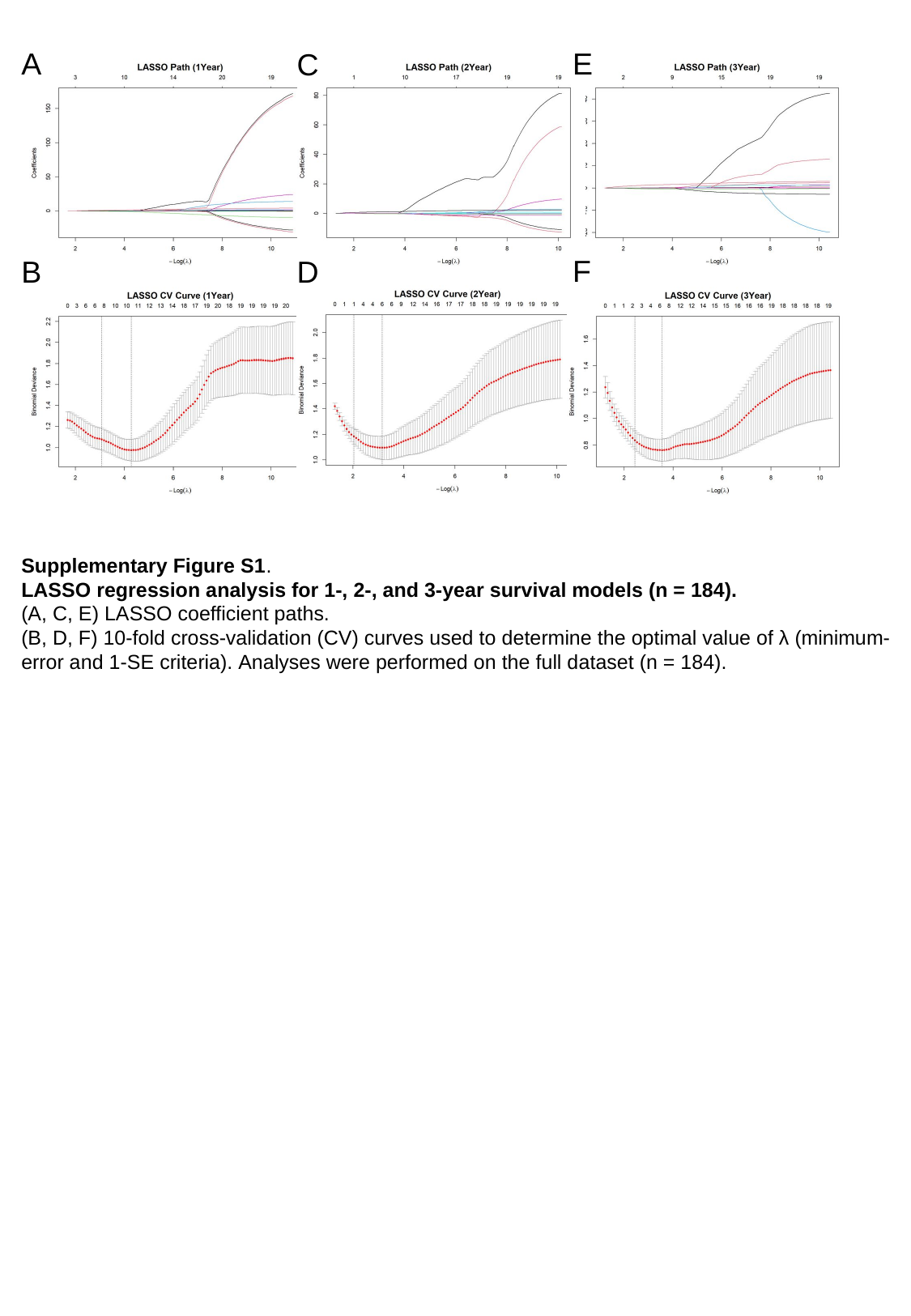

E
A
C
F
B
D
Supplementary Figure S1.
LASSO regression analysis for 1-, 2-, and 3-year survival models (n = 184).
(A, C, E) LASSO coefficient paths.
(B, D, F) 10-fold cross-validation (CV) curves used to determine the optimal value of λ (minimum-error and 1-SE criteria). Analyses were performed on the full dataset (n = 184).

## Slide 2
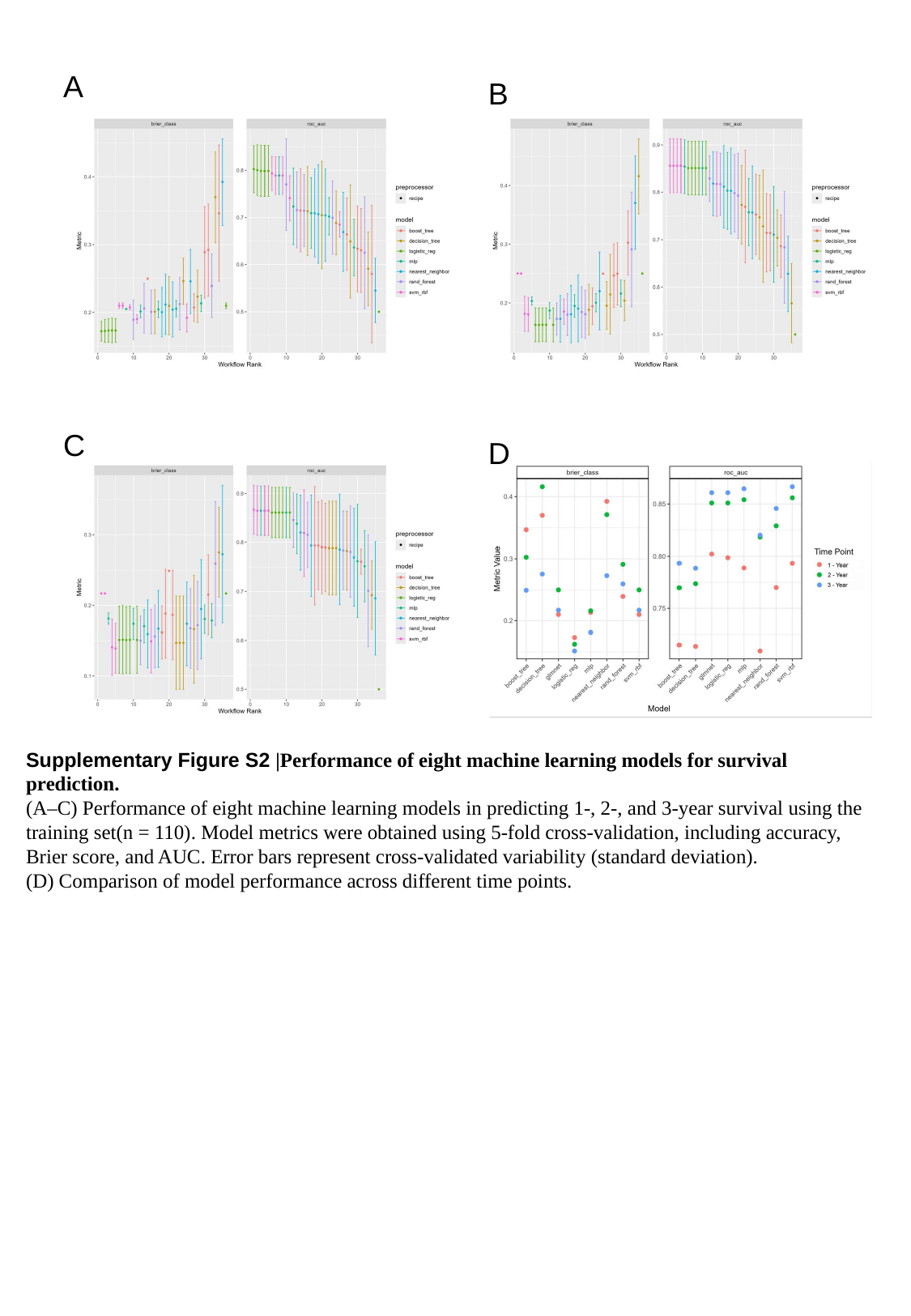

A
B
C
D
Supplementary Figure S2 |Performance of eight machine learning models for survival prediction.
(A–C) Performance of eight machine learning models in predicting 1-, 2-, and 3-year survival using the training set(n = 110). Model metrics were obtained using 5-fold cross-validation, including accuracy, Brier score, and AUC. Error bars represent cross-validated variability (standard deviation).
(D) Comparison of model performance across different time points.

## Slide 3
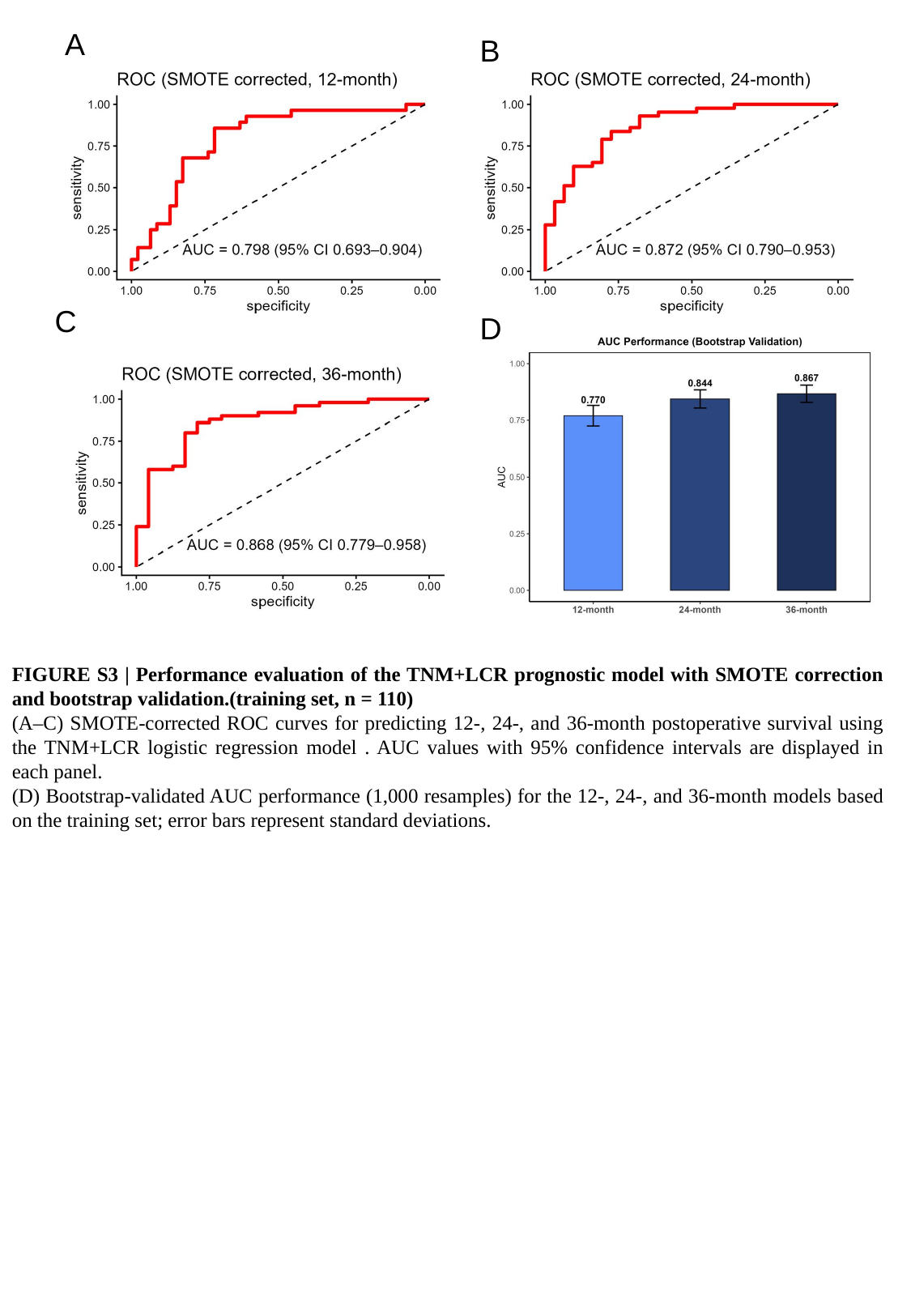

A
B
C
D
FIGURE S3 | Performance evaluation of the TNM+LCR prognostic model with SMOTE correction and bootstrap validation.(training set, n = 110)
(A–C) SMOTE-corrected ROC curves for predicting 12-, 24-, and 36-month postoperative survival using the TNM+LCR logistic regression model . AUC values with 95% confidence intervals are displayed in each panel.
(D) Bootstrap-validated AUC performance (1,000 resamples) for the 12-, 24-, and 36-month models based on the training set; error bars represent standard deviations.
