# Supplementary material for: Machine Learning–Driven Prognostic Model Integrating Lymphocyte‐to‐C‐Reactive Protein Ratio and TNM Staging in Gallbladder Cancer
Source: Cancer Med. 2026 Feb 20;15(3):e71646. doi: 10.1002/cam4.71646 (PMC12928047; doi:10.1002/cam4.71646)
Supplement: Supplementary file 3 — Data S3: Supporting Information. [file CAM4-15-e71646-s003.docx]

#### Supplementary Code S1 ####

# Data preprocessing, TNM classification, mGPS calculation,

# statistical utilities, and ROC plotting function

#### 0. Load required libraries ################################

library(tidyverse)

library(pROC)

library(nortest)

library(writexl)

library(glmnet)

library(caret)

library(CBCgrps)

library(ggpubr)

library(rms)

library(readxl)

#### Section 1: Utility Functions #############################

##### 1.1 ROC curve plotting with AUC + sensitivity + specificity #####

save_roc_plot <- function(roc_obj, output_file, survival_time) {

auc_value <- auc(roc_obj)

th <- coords(

roc_obj, "best",

ret = c("threshold", "sensitivity", "specificity")

)

best_threshold <- th["threshold"]

sensitivity <- th["sensitivity"]

specificity <- th["specificity"]

print(paste("AUC:", auc_value))

print(paste("Best threshold:", best_threshold))

print(paste("Sensitivity:", sensitivity))

print(paste("Specificity:", specificity))

g <- ggroc(

roc_obj,

alpha = 1,

size = 0.8,

legacy.axes = TRUE,

color = "red"

) +

theme_classic2() +

ggtitle("ROC Curve") +

annotate("segment", x = 0, y = 0, xend = 1, yend = 1) +

annotate("text", x = 0.75, y = 0.25,

label = paste("AUC:", round(auc_value, 3)),

size = 4) +

annotate("text", x = 0.75, y = 0.20,

label = paste("Sensitivity:", round(sensitivity, 3)),

size = 4) +

annotate("text", x = 0.75, y = 0.15,

label = paste("Specificity:", round(specificity, 3)),

size = 4)

ggsave(output_file, g, width = 8, height = 6, dpi = 300)

}

#### Section 2: TNM Classification Functions ##################

##### 2.1 Simplified TNM classification #####

get_stage_simple <- function(T, N, M) {

if (M == 1 | N == 2) return(4)

if (T == 1 & N == 0) return(1)

if (T == 2 & N == 0) return(2)

if (T == 3 & N == 0) return(3)

if (T %in% c(1, 2, 3) & N == 1) return(3)

if (T == 4 & N %in% c(0, 1)) return(4)

return(NA)

}

##### 2.2 Vectorized version #####

get_stage_simple_vec <- Vectorize(get_stage_simple)

#### Section 3: mGPS Calculation ##############################

##### 3.1 mGPS function #####

get_mGPS <- function(CRP, ALB) {

stopifnot(length(CRP) == length(ALB))

score <- rep(NA_integer_, length(CRP))

score[CRP <= 10] <- 0

score[CRP > 10 & ALB >= 35] <- 1

score[CRP > 10 & ALB < 35] <- 2

return(score)

}

#### Section 4: Data Loading & Preprocessing ###################

##### 4.1 Load raw data #####

data <- read.csv("data.csv", stringsAsFactors = FALSE)

##### 4.2 Missing-value handling #####

data <- data %>% drop_na()

##### 4.3 Convert numeric variables #####

num_cols <- c("NEU", "L", "plt", "WBC", "CRP", "ALB")

data[num_cols] <- lapply(data[num_cols], function(x) as.numeric(as.character(x)))

data <- data %>%

mutate(

diagnosis12 = ifelse(Time <= 12 & Status == 1, 1, 0),

diagnosis24 = ifelse(Time <= 24 & Status == 1, 1, 0),

diagnosis36 = ifelse(Time <= 36 & Status == 1, 1, 0)

)

#### Section 5: Derived Inflammatory / Nutritional Markers ####

##### 5.1 mGPS #####

data$mGPS <- mapply(get_mGPS, data$CRP, data$ALB)

##### 5.2 Compute ratios #####

data$NLR <- data$NEU / data$L

data$PLR <- data$plt / data$L

data$LWR <- data$L / data$WBC

data$NWR <- data$NEU / data$WBC

data$LCR <- data$L / data$CRP

data$CAR <- data$CRP / data$ALB

data$SII <- data$plt * data$NEU / data$L

data$PNI <- data$ALB + 5 * data$L

##### 5.3 TNM stage #####

data$T <- as.integer(data$T)

data$N <- as.integer(data$N)

if (!("M" %in% names(data)) || length(data$M) != nrow(data)) {

data$M <- 0

}

data$M <- as.integer(data$M)

data$TNM <- get_stage_simple_vec(data$T, data$N, data$M)

##### 5.4 Save output #####

write.csv(data, "data_processed.csv", row.names = FALSE)

#### End of Supplementary Code S1 #############################

#### Supplementary Code S2: Univariate & Multivariate Cox Regression ####

#### 0. Load libraries ####

library(survival)

library(dplyr)

library(broom)

library(purrr)

#### 1. Load data & prepare survival object ####

data <- read.csv("data_processed.csv", stringsAsFactors = FALSE)

# Convert to factors

data$Sex <- factor(data$Sex, levels = c(1,0), labels = c("Male","Female"))

data$TNM <- factor(data$TNM)

data$T <- factor(data$T)

data$N <- factor(data$N)

data$mGPS <- factor(data$mGPS)

# Survival object

surv_obj <- with(data, Surv(Time, Status))

#### 2. Univariate Cox regression ####

##### 2.1 Function for univariate Cox #####

fit_univ <- function(var){

fml <- as.formula(sprintf("surv_obj ~ `%s`", var))

fit <- coxph(fml, data = data, ties = "efron", na.action = na.exclude)

tt <- broom::tidy(fit, exponentiate = TRUE, conf.int = TRUE)

# --- Factor variable ---

if (is.factor(data[[var]])) {

level_from_term <- sub(sprintf("^`?%s`?", var), "", tt$term)

tt$Level <- level_from_term

ref <- levels(data[[var]])[1]

ref_row <- tibble(

Variable = var,

Level = ref,

HR = 1,

LCL = NA_real_,

UCL = NA_real_,

P.value = NA_real_

)

out <- tt %>%

transmute(

Variable = var,

Level,

HR = estimate,

LCL = conf.low,

UCL = conf.high,

P.value = p.value

) %>%

bind_rows(ref_row, .) %>%

mutate(

`Hazard Ratio (95%CI)` = ifelse(

is.na(LCL),

"1",

sprintf("%.3f (%.3f–%.3f)", HR, LCL, UCL)

),

`P-value` = ifelse(is.na(P.value), "",

ifelse(P.value < 0.001, "<0.001", sprintf("%.3f", P.value)))

) %>%

select(Variable, Level, `Hazard Ratio (95%CI)`, `P-value`)

} else {

# --- Continuous variable ---

out <- tt %>%

transmute(

Variable = var,

Level = "(continuous)",

HR = estimate,

LCL = conf.low,

UCL = conf.high,

P.value = p.value

) %>%

mutate(

`Hazard Ratio (95%CI)` = sprintf("%.3f (%.3f–%.3f)", HR, LCL, UCL),

`P-value` = ifelse(P.value < 0.001, "<0.001", sprintf("%.3f", P.value))

) %>%

select(Variable, Level, `Hazard Ratio (95%CI)`, `P-value`)

}

out

}

##### 2.2 Variables to test #####

vars <- c(

"Age","Sex","TB","CRP","ALB","Hb","plt","WBC","NEU","L",

"NLR","PLR","LWR","NWR","LCR","CAR","PNI",

"T","N","mGPS","TNM"

)

##### 2.3 Run all univariate Cox #####

univ_tbl <- purrr::map_dfr(vars, fit_univ)

#### 3. Multivariate Cox regression ####

##### 3.1 Choose variables for multivariate model #####

multi_vars <- c("TB","CRP","ALB","Hb","WBC","NEU","NLR","PLR",

"LWR","NWR","LCR","CAR","PNI","TNM","mGPS")

##### 3.2 Fit multivariate Cox #####

multi_fml <- as.formula(

paste("surv_obj ~", paste(sprintf("`%s`", multi_vars), collapse = " + "))

)

multi_fit <- coxph(multi_fml, data = data, ties = "efron", na.action = na.exclude)

##### 3.3 Format multivariate output #####

make_multi_table <- function(fit, data){

tb <- broom::tidy(fit, exponentiate = TRUE, conf.int = TRUE)

tb <- tb %>%

mutate(

Variable = sub("=.*$", "", term),

Level = ifelse(grepl("=", term),

sub("^.*?=", "", term),

"(continuous)")

) %>%

transmute(

Variable, Level,

HR = estimate, LCL = conf.low, UCL = conf.high, P.value = p.value

) %>%

mutate(

`Hazard Ratio (95%CI)` = sprintf("%.3f (%.3f–%.3f)", HR, LCL, UCL),

`P-value` = ifelse(P.value < 0.001, "<0.001", sprintf("%.3f", P.value))

) %>%

select(Variable, Level, `Hazard Ratio (95%CI)`, `P-value`)

# Add reference rows for factor variables

facs <- names(Filter(is.factor, data[multi_vars]))

ref_rows <- purrr::map_dfr(facs, function(v){

tibble(

Variable = v,

Level = levels(data[[v]])[1],

`Hazard Ratio (95%CI)` = "1",

`P-value` = ""

)

})

out <- bind_rows(ref_rows %>% filter(Variable %in% unique(tb$Variable)),

tb)

out

}

multi_tbl <- make_multi_table(multi_fit, data)

#### End of Supplementary Code S2 ####

#### Supplementary Code S3: LASSO Logistic Regression ####

#### 0. Load libraries ####

library(glmnet)

library(dplyr)

library(pROC)

library(ggplot2)

#### 1. Candidate predictors ####

predictors <- c(

"TB","CRP","ALB","Hb","plt","WBC","NEU","L",

"CEA","CA125","NLR","PLR","LWR","NWR","CAR",

"LCR","SII","PNI","mGPS","TNM"

)

# Convert necessary categorical variables

data$Sex <- factor(data$Sex, levels=c(0,1), labels=c("Female","Male"))

#### 2. LASSO fitting function (for diagnosis12/24/36) ####

fit_lasso_binom <- function(outcome){

df <- data %>%

select(all_of(c(outcome, predictors))) %>%

na.omit()

# response 0/1

y <- as.integer(df[[outcome]])

# one-hot encoding for factor predictors

X <- model.matrix(reformulate(predictors), data=df)[, -1]

# LASSO with 10-fold CV

set.seed(123)

cvfit <- cv.glmnet(

X, y,

family="binomial",

alpha=1,

nfolds=10

)

# lambda selection

lam_min <- cvfit$lambda.min

lam_1se <- cvfit$lambda.1se

# nonzero coefficients at lambda.1se

coefs <- coef(cvfit, s="lambda.1se")

nz_idx <- which(as.numeric(coefs) != 0)

sel <- data.frame(

Feature = rownames(coefs)[nz_idx],

Coef = as.numeric(coefs)[nz_idx],

row.names = NULL

)

# AUC (train performance)

p_hat <- as.numeric(predict(

cvfit, newx=X, s="lambda.1se", type="response"

))

auc_val <- as.numeric(roc(y, p_hat)$auc)

list(

cvfit=cvfit,

lambda.min=lam_min,

lambda.1se=lam_1se,

selected=sel,

auc=auc_val,

X=X,

y=y

)

}

#### 3. Run LASSO for 12/24/36-month mortality ####

fit12 <- fit_lasso_binom("diagnosis12")

fit24 <- fit_lasso_binom("diagnosis24")

fit36 <- fit_lasso_binom("diagnosis36")

# 查看 12-month 结果

fit12$lambda.1se

fit12$auc

subset(fit12$selected, Feature != "(Intercept)")

#### 4. Extract selected predictors ####

sel12 <- subset(fit12$selected, Feature != "(Intercept)")

sel24 <- subset(fit24$selected, Feature != "(Intercept)")

sel36 <- subset(fit36$selected, Feature != "(Intercept)")

list(sel12=sel12, sel24=sel24, sel36=sel36)

#### 5. Plot CV curves & coefficient paths ####

##### 5.1 Save both CV curve and coefficient path #####

save_lasso_plots <- function(fit, tag){

# CV curve

png(paste0("LASSO_CV_", tag, ".png"),

width=1600, height=1200, res=200)

plot(fit$cvfit)

title(main=paste0("LASSO CV Curve (", tag, ")"),

line=2.5, cex.main=1.5)

dev.off()

# Coefficient path

png(paste0("LASSO_Path_", tag, ".png"),

width=1600, height=1200, res=200)

plot(fit$cvfit$glmnet.fit, xvar="lambda", label=FALSE)

title(main=paste0("LASSO Path (", tag, ")"),

line=2.5, cex.main=1.5)

dev.off()

}

##### 5.2 Save plots for 12/24/36 models #####

save_lasso_plots(fit12, "1Year")

save_lasso_plots(fit24, "2Year")

save_lasso_plots(fit36, "3Year")

#### End of Supplementary Code S3 ####

#### Supplementary Code S4: Baseline table & AUC ranking ######

###### 0. 前期准备：加载包、读入数据 ######

library(writexl)

library(corrplot)

library(glmnet)

library(caret)

library(CBCgrps) # twogrps() 来自该包

library(nortest)

library(tidyverse)

library(ggpubr)

library(rms)

library(pROC)

library(dplyr)

library(readxl)

library(ggplot2)

library(viridis)

# 数据整理：主要靠 Excel，导入后做轻微设置改动

# 投稿时不要保留本地路径，例如 D:/、G:/ 等

data_raw <- read.csv("data.csv", stringsAsFactors = FALSE)

data <- na.omit(data_raw)

# 构建 12/24/36 月死亡标签

data <- data %>%

mutate(

diagnosis12 = ifelse(Time <= 12 & Status == 1, 1, 0),

diagnosis24 = ifelse(Time <= 24 & Status == 1, 1, 0),

diagnosis36 = ifelse(Time <= 36 & Status == 1, 1, 0)

)

# 设置因子

data$Sex <- as.factor(data$Sex)

data$diagnosis12 <- as.factor(data$diagnosis12)

data$diagnosis24 <- as.factor(data$diagnosis24)

data$diagnosis36 <- as.factor(data$diagnosis36)

# 有时候 twogrps 需要 data.frame

data <- as.data.frame(data)

###### 1. 基线表（以 36 月是否死亡为分组变量） ######

# CBCgrps::twogrps

tab1 <- twogrps(data, gvar = "diagnosis36")

# 如果导出报错，可先： tab1 <- as.data.frame(tab1$Table)

write.csv(tab1$Table, file = "Baseline_diagnosis36.csv", row.names = FALSE)

###### 2. AUC 排序分析（12 / 24 / 36 月生存） ######

##### 2.1 构建 ROC 使用的数据 #####

rocdata <- data %>%

mutate(

Sex = as.numeric(Sex) # 因子转数值用于模型拟合

)

# 根据你原始脚本中用到的变量列出预测指标

predictors_auc <- c(

"Sex","Age","TB","CRP","ALB","Hb","plt","WBC",

"NEU","L","CA199","CEA","CA125","T","N",

"NLR","PLR","LWR","NWR","LCR","CAR","SII",

"PNI","mGPS","TNM"

)

##### 2.2 通用函数：计算某个结局的多变量 ROC + 导出 AUC 条形图 #####

run_auc_bar <- function(outcome, time_label) {

# outcome 例如 "diagnosis12"

# time_label 例如 "12"、"24"、"36"

# 构建公式： diagnosisXX ~ Sex + Age + ...

fml <- as.formula(

paste(outcome, "~", paste(predictors_auc, collapse = "+"))

)

# roc.list 是一个 list，每个元素对应一个 predictor 的 ROC

roc.list <- roc(fml, data = rocdata)

# 提取 AUC 值（属性索引 9）

auc_vec <- sapply(roc.list, "[", 9)

auc_df <- data.frame(

Variable = names(auc_vec),

AUC = as.numeric(auc_vec),

row.names = NULL

) %>%

arrange(desc(AUC))

# 可选：去掉变量名中的 ".auc" 后缀

auc_df$Variable <- gsub("\\.auc$", "", auc_df$Variable)

# 画条形图

p <- ggplot(auc_df,

aes(x = AUC,

y = reorder(Variable, AUC),

fill = Variable)) +

geom_col(width = 0.7, color = "black") +

geom_text(aes(label = sprintf("%.2f", AUC)),

hjust = -0.2, size = 3.5) +

scale_fill_viridis_d(option = "E") +

labs(

x = "AUC",

y = "Variable",

title = paste0("AUC (", time_label, "-Year Survival)")

) +

scale_x_continuous(

limits = c(0, max(auc_df$AUC) + 0.1),

expand = c(0, 0)

) +

theme_bw(base_size = 14) +

theme(

legend.position = "none",

plot.title = element_text(hjust = 0.5, face = "bold"),

panel.border = element_rect(color = "black", fill = NA)

)

# 保存图（PNG + PDF）

ggsave(paste0("AUC_barplot_", time_label, "y.png"),

plot = p, width = 5, height = 6, dpi = 300)

ggsave(paste0("AUC_barplot_", time_label, "y.pdf"),

plot = p, width = 5, height = 6)

# 导出 AUC 表

write.csv(auc_df,

paste0("AUC_", time_label, "y.csv"),

row.names = FALSE)

# 返回对象，如果需要在 R 里查看

list(roc.list = roc.list, auc = auc_df, plot = p)

}

##### 2.3 分别运行 12 / 24 / 36 月 #####

res_auc_12 <- run_auc_bar("diagnosis12", "1") # 1-Year

res_auc_24 <- run_auc_bar("diagnosis24", "2") # 2-Year

res_auc_36 <- run_auc_bar("diagnosis36", "3") # 3-Year

###### End of Supplementary Code S4 ######

#### Supplementary Code S5: Machine Learning Models and Hyperparameter Tuning ####

##### 1. Load Required Libraries #####

library(tidymodels)

library(finetune)

library(tidyverse)

library(ggplot2)

library(openxlsx)

library(gt)

library(stringr)

##### 2. Load Training Data #####

train <- read.csv("train.csv")

train$diagnosis12 <- as.factor(train$diagnosis12)

train$diagnosis24 <- as.factor(train$diagnosis24)

train$diagnosis36 <- as.factor(train$diagnosis36)

##### 3. Define Function for 3 Time Points #####

run_ml <- function(train, target){

set.seed(2025)

folds <- vfold_cv(train, v = 5, strata = !!sym(target))

rec <- recipe(as.formula(paste0(target, " ~ LCR + TNM")), data=train) %>%

step_normalize(all_predictors())

models <- list(

logistic_reg = logistic_reg() %>%

set_engine("glm") %>%

set_mode("classification"),

decision_tree = decision_tree(

cost_complexity = tune(),

min_n = tune()

) %>%

set_engine("rpart") %>%

set_mode("classification"),

boost_tree = boost_tree(

trees = tune(),

learn_rate = tune()

) %>%

set_engine("xgboost") %>%

set_mode("classification"),

rand_forest = rand_forest(

trees = tune(),

mtry = tune(),

min_n = tune()

) %>%

set_engine("ranger") %>%

set_mode("classification"),

svm_rbf = svm_rbf(

cost = tune(),

rbf_sigma = tune()

) %>%

set_engine("kernlab") %>%

set_mode("classification"),

nearest_neighbor = nearest_neighbor(

neighbors = tune()

) %>%

set_engine("kknn") %>%

set_mode("classification"),

glmnet = logistic_reg(

penalty = tune(),

mixture = tune()

) %>%

set_engine("glmnet") %>%

set_mode("classification"),

mlp = mlp(

hidden_units = tune(),

penalty = tune()

) %>%

set_engine("nnet") %>%

set_mode("classification")

)

wf_set <- workflow_set(

preproc = list(recipe = rec),

models = models,

cross = TRUE

)

metrics2 <- metric_set(

roc_auc,

brier_class

)

set.seed(2024)

res <- wf_set %>%

workflow_map(

resamples = folds,

grid = 5,

metrics = metrics2,

control = control_race(verbose = TRUE)

)

p <- autoplot(res)

ggsave(paste0("ML_compare_", target, ".png"), p, width = 9, height = 6, dpi = 300)

best_all <- collect_metrics(res) %>%

group_by(wflow_id, .metric) %>%

arrange(desc(mean)) %>%

slice(1) %>%

ungroup() %>%

select(wflow_id, .metric, mean) %>%

pivot_wider(names_from = .metric, values_from = mean)

best_all$time <- target

models2 <- unique(res$wflow_id)

best_param_list <- list()

for (m in models2) {

res_m <- extract_workflow_set_result(res, m)

best_param_list[[m]] <- select_best(res_m, metric = "roc_auc")

}

best_table <- map_df(names(best_param_list), function(nm) {

df <- best_param_list[[nm]]

df$Model <- nm

df

}) %>% relocate(Model)

write.xlsx(best_table, file = paste0("Best_Model_Params_", target, ".xlsx"), rowNames = FALSE)

return(list(res=res, best_all=best_all, best_param=best_param_list))

}

##### 4. Run ML for 12/24/36 Months #####

res12 <- run_ml(train, "diagnosis12")

res24 <- run_ml(train, "diagnosis24")

res36 <- run_ml(train, "diagnosis36")

##### 5. Merge Summary Results #####

best_all12 <- res12$best_all

best_all24 <- res24$best_all

best_all36 <- res36$best_all

all_results <- bind_rows(best_all12, best_all24, best_all36)

##### 6. Long Format for Visualization #####

all_long <- all_results %>%

pivot_longer(cols = c(brier_class, roc_auc),

names_to = "metric",

values_to = "value") %>%

mutate(wflow_id = str_remove(wflow_id, "^recipe_"))

##### 7. Combined ML Visualization #####

p <- ggplot(all_long, aes(x = wflow_id, y = value, color = time)) +

geom_point(size = 3) +

facet_wrap(~ metric, scales = "free_y") +

theme_bw(base_size = 14) +

labs(

x = "Model",

y = "Metric Value",

color = "Time Point"

) +

theme(

panel.border = element_rect(color = "black", fill = NA, linewidth = 0.9),

strip.background = element_rect(color = "black", fill = "white", linewidth = 0.9),

axis.text.x = element_text(angle = 45, hjust = 1)

)

ggsave("ML_compare_all_months.png", p, width = 9, height = 6, dpi = 300)

#### Supplementary Code S5: Logistic Regression, Nomogram, ROC, Calibration, and Bootstrap Validation ####

##### 1. Load Packages and Data #####

library(writexl)

library(corrplot)

library(glmnet)

library(caret)

library(CBCgrps)

library(nortest)

library(tidyverse)

library(ggpubr)

library(rms)

library(pROC)

library(dplyr)

library(readxl)

library(ggplot2)

# 请在运行前设置工作目录，并确保 data.csv / train.csv / test.csv 存在

# setwd("your_path_here")

data_raw <- read.csv("data.csv")

data <- na.omit(data_raw)

# 构建 1/2/3 年随访结局（二分类）

data <- data %>%

mutate(

diagnosis12 = ifelse(Time <= 12 & Status == 1, 1, 0),

diagnosis24 = ifelse(Time <= 24 & Status == 1, 1, 0),

diagnosis36 = ifelse(Time <= 36 & Status == 1, 1, 0)

)

data$Sex <- as.factor(data$Sex)

data$diagnosis12 <- as.factor(data$diagnosis12)

data$diagnosis24 <- as.factor(data$diagnosis24)

data$diagnosis36 <- as.factor(data$diagnosis36)

##### 2. Train/Test Split (if you need to re-generate) #####

## 如已手动生成 train.csv / test.csv，可注释掉本段

# set.seed(12)

# train_id <- sample(1:nrow(data), 0.6 * nrow(data))

# train <- data[train_id, ]

# test <- data[-train_id, ]

# write.csv(train, "train.csv", row.names = FALSE)

# write.csv(test, "test.csv", row.names = FALSE)

train <- read.csv("train.csv")

test <- read.csv("test.csv")

##### 3. Utility Function: Fit Logistic + Nomogram + ROC + Calibration #####

run_nomogram_block <- function(train, test, outcome, time_label){

# 3.1 rms 设置

dd <- datadist(train)

options(datadist = "dd")

# 3.2 lrm 模型（用于列线图）

fml_lrm <- as.formula(paste0(outcome, " ~ LCR + TNM"))

fit_lrm <- lrm(fml_lrm, data = train, x = TRUE, y = TRUE)

print(fit_lrm)

# 3.3 列线图并保存

nom <- nomogram(

fit_lrm,

fun = plogis,

fun.at = c(0.001, 0.01, 0.05, 0.5, 0.95, 0.99, 0.999),

lp = TRUE,

funlabel = "diagnosis rate"

)

png(paste0("nomogram_", time_label, ".png"),

width = 2400, height = 1800, res = 300)

plot(nom, xfrac = 0.35)

dev.off()

# 3.4 logistic 回归（base glm，用于预测）

fit_glm <- glm(fml_lrm, data = train, family = binomial(link = "logit"))

summary(fit_glm)

# 3.5 训练集/验证集预测

p_train <- predict(fit_glm, newdata = train, type = "response")

p_test <- predict(fit_glm, newdata = test, type = "response")

y_train <- train[[outcome]]

y_test <- test[[outcome]]

# 3.6 ROC：训练集

roc_train <- roc(y_train, p_train)

auc_tr <- as.numeric(auc(roc_train))

ci_tr <- ci.auc(roc_train)

lab_tr <- sprintf("AUC = %.3f (95%% CI %.3f–%.3f)",

auc_tr, ci_tr[1], ci_tr[3])

g_tr <- ggroc(roc_train, alpha = 1, size = 0.8,

legacy.axes = TRUE, color = "red")

p_tr <- g_tr +

theme_classic2() +

ggtitle(paste0("Train_", time_label)) +

annotate("segment", x = 1, y = 1, xend = 0, yend = 0,

linetype = "dashed") +

annotate("text", x = 0.98, y = 0.02, label = lab_tr,

hjust = 1, vjust = 0, size = 4)

ggsave(paste0("ROC_train_", time_label, ".png"),

plot = p_tr, width = 1200, height = 800,

units = "px", dpi = 300)

# 3.7 ROC：验证集

roc_te <- roc(y_test, p_test)

auc_te <- as.numeric(auc(roc_te))

ci_te <- ci.auc(roc_te)

lab_te <- sprintf("AUC = %.3f (95%% CI %.3f–%.3f)",

auc_te, ci_te[1], ci_te[3])

g_te <- ggroc(roc_te, alpha = 1, size = 0.8,

legacy.axes = TRUE, color = "red")

p_te <- g_te +

theme_classic2() +

ggtitle(paste0("Test_", time_label)) +

annotate("segment", x = 1, y = 1, xend = 0, yend = 0,

linetype = "dashed") +

annotate("text", x = 0.98, y = 0.02, label = lab_te,

hjust = 1, vjust = 0, size = 4)

ggsave(paste0("ROC_test_", time_label, ".png"),

plot = p_te, width = 1200, height = 800,

units = "px", dpi = 300)

# 3.8 校正曲线：训练集（rms::calibrate）

cal_tr <- calibrate(fit_lrm,

cmethod = "hare",

method = "boot",

B = 1000,

xlab = "Nomogram Predicted Probability",

ylab = "Observed Probability")

png(paste0("Calib_train_", time_label, ".png"),

width = 1600, height = 1200, res = 300)

plot(cal_tr,

xlim = c(0, 1.0), ylim = c(0, 1.0),

xlab = "Nomogram Predicted Probability",

ylab = "Observed Probability")

dev.off()

# 3.9 校正曲线：验证集（先用 phat，再用 lrm + calibrate）

test_tmp <- test

test_tmp$phat <- p_test

fit_cal <- lrm(as.formula(paste0(outcome, " ~ phat")),

data = test_tmp, x = TRUE, y = TRUE)

cal_te <- calibrate(fit_cal, method = "boot", B = 1000)

png(paste0("Calib_test_", time_label, ".png"),

width = 1600, height = 1200, res = 300)

plot(cal_te,

xlim = c(0, 1.0), ylim = c(0, 1.0),

xlab = "Nomogram Predicted Probability",

ylab = "Observed Probability")

dev.off()

list(

fit_lrm = fit_lrm,

fit_glm = fit_glm,

roc_train = roc_train,

roc_test = roc_te

)

}

##### 4. Run for 12/24/36 Months #####

dd <<- datadist(train) # 用 <<- 明确放到 global environment

options(datadist = "dd")

res_12 <- run_nomogram_block(train, test,

outcome = "diagnosis12",

time_label = "12m")

res_24 <- run_nomogram_block(train, test,

outcome = "diagnosis24",

time_label = "24m")

res_36 <- run_nomogram_block(train, test,

outcome = "diagnosis36",

time_label = "36m")

##### 5. Bootstrap AUC Validation #####

bootstrap_auc <- function(data, outcome, n_boot = 1000){

boot_auc <- numeric(n_boot)

for (i in seq_len(n_boot)) {

idx <- sample(1:nrow(data), replace = TRUE)

train <- data[idx, ]

test <- data[-unique(idx), ]

fml <- as.formula(paste0(outcome, " ~ LCR + TNM"))

fit <- glm(fml, data = train, family = "binomial")

pred <- predict(fit, newdata = test, type = "response")

boot_auc[i] <- auc(roc(test[[outcome]], pred))

}

c(mean = mean(boot_auc), sd = sd(boot_auc))

}

data_full <- read.csv("data.csv") # 保证有 diagnosis12/24/36 列

data_full <- na.omit(data_full)

auc12 <- bootstrap_auc(data_full, "diagnosis12", n_boot = 1000)

auc24 <- bootstrap_auc(data_full, "diagnosis24", n_boot = 1000)

auc36 <- bootstrap_auc(data_full, "diagnosis36", n_boot = 1000)

##### 6. Bar Plot of Bootstrap AUC #####

df_auc <- data.frame(

Time = factor(c("12-month", "24-month", "36-month"),

levels = c("12-month", "24-month", "36-month")),

AUC = c(auc12["mean"], auc24["mean"], auc36["mean"]),

SD = c(auc12["sd"], auc24["sd"], auc36["sd"])

)

g_auc <- ggplot(df_auc, aes(x = Time, y = AUC, fill = AUC)) +

geom_col(width = 0.55, color = "black", linewidth = 0.5) +

geom_errorbar(aes(ymin = AUC - SD, ymax = AUC + SD),

width = 0.12, size = 0.9, color = "black") +

geom_text(aes(label = sprintf("%.3f", AUC)),

vjust = -1.8, size = 5.2, fontface = "bold") +

scale_fill_gradient(low = "#5B8FF9", high = "#1E315B") +

theme_bw(base_size = 15) +

theme(

panel.grid = element_blank(),

legend.position = "none",

axis.line = element_line(color = "black", linewidth = 0.8),

panel.border = element_rect(color = "black", fill = NA, linewidth = 1),

axis.title.x = element_blank(),

axis.text.x = element_text(face = "bold", size = 14),

axis.text.y = element_text(size = 12),

plot.title = element_text(face = "bold", size = 16, hjust = 0.5)

) +

ylim(0, 1) +

labs(

title = "AUC Performance (Bootstrap Validation)",

y = "AUC"

)

ggsave("Bootstrap_AUC_barplot.png", g_auc,

width = 8, height = 6, dpi = 300)

#### SMOTE 校正与 ROC 分析（一级标题）####

##### SMOTE 构建函数（可直接运行）（二级标题）#####

library(pROC)

library(ggplot2)

library(smotefamily)

build_smote_roc <- function(train, test, timepoint = "12") {

# 目标列名称

target_col <- paste0("diagnosis", timepoint)

# 训练数据，仅选 LCR、TNM、标签

df_train <- train[, c("LCR", "TNM", target_col)]

# SMOTE 要求数值型

df_train$LCR <- as.numeric(df_train$LCR)

df_train$TNM <- as.numeric(df_train$TNM)

df_train[[target_col]] <- as.numeric(as.character(df_train[[target_col]]))

# ① SMOTE 重采样

smote_res <- SMOTE(

X = df_train[, c("LCR", "TNM")],

target = df_train[[target_col]],

K = 5,

dup_size = 1

)

# 构建 SMOTE 后数据集

train_smote <- data.frame(

LCR = smote_res$data$LCR,

TNM = smote_res$data$TNM,

y = as.factor(smote_res$data$class)

)

# ② 拟合 Logistic 回归

fit <- glm(y ~ LCR + TNM, data = train_smote, family = "binomial")

# ③ 预测

pred <- predict(fit, newdata = test, type = "response")

roc_obj <- roc(test[[target_col]], pred)

auc_val <- as.numeric(auc(roc_obj))

ci_val <- ci.auc(roc_obj)

auc_label <- sprintf(

"AUC = %.3f (95%% CI %.3f–%.3f)",

auc_val, ci_val[1], ci_val[3]

)

# ④ 绘图

p <- ggroc(roc_obj, size = 1.2, color = "red") +

theme_classic() +

ggtitle(paste0("ROC (SMOTE corrected, ", timepoint, "-month)")) +

annotate("segment", x = 0, y = 1, xend = 1, yend = 0, linetype = "dashed") +

annotate("text", x = 0.01, y = 0.1, label = auc_label,

hjust = 1, vjust = 0, size = 4)

# ⑤ 保存图像

fname <- paste0("ROC_", timepoint, "month_SMOTE.png")

ggsave(fname, p, width = 4, height = 2.67, dpi = 300)

message("Saved: ", fname)

return(list(model = fit, roc = roc_obj, plot = p))

}

##### 分别计算 12、24、36 月 ROC #####

res12 <- build_smote_roc(train, test, "12")

res24 <- build_smote_roc(train, test, "24")

res36 <- build_smote_roc(train, test, "36")

#### Supplementary Code S6 — Decision Curve Analysis (DCA) ####

#### Automatic time-point title; display only, no saving ####

library(rmda)

library(ggplot2)

library(grid)

# Function: run_dca_nosave()

# Description:

# • Compute DCA on training and testing sets

# • Outcome: diagnosis{timepoint} (e.g., diagnosis12)

# • Model: logistic regression (LCR + TNM)

# • Automatically adds time-title: "12-month", "24-month", etc.

# • Returns plotted DCA curve but does NOT save files

run_dca_nosave <- function(train, test, tp = "12") {

outcome_col <- paste0("diagnosis", tp)

title_text <- paste0(tp, "-month Decision Curve Analysis")

#### Ensure binary outcome is numeric (0/1) ####

t1 <- train

t2 <- test

t1$diagnosis <- as.numeric(as.character(t1[[outcome_col]]))

t2$diagnosis <- as.numeric(as.character(t2[[outcome_col]]))

#### Train-set DCA ####

dca_train <- decision_curve(

diagnosis ~ LCR + TNM,

data = t1, family = binomial(link="logit"),

thresholds = seq(0,1,0.01),

confidence.intervals = FALSE,

study.design = "case-control",

population.prevalence = 0.3

)

#### Test-set DCA ####

dca_test <- decision_curve(

diagnosis ~ LCR + TNM,

data = t2, family = binomial(link="logit"),

thresholds = seq(0,1,0.01),

confidence.intervals = FALSE,

study.design = "case-control",

population.prevalence = 0.3

)

#### Base DCA plot ####

p_base <- plot_decision_curve(

list(dca_test, dca_train),

curve.names = c("Test", "Train"),

col = c("red", "blue"),

standardize = FALSE,

cost.benefit.axis = FALSE

)

#### Add time-point title on top ####

grid.text(

title_text,

x = 0.5, y = 0.96,

gp = gpar(fontsize = 14, fontface = "bold")

)

return(invisible(p_base))

}

#### Run DCA (12m / 24m / 36m) — Display only ####

p12 <- run_dca_nosave(train, test, "12")

p24 <- run_dca_nosave(train, test, "24")

p36 <- run_dca_nosave(train, test, "36")

#### Supplementary Code S7 — Temporal Stability of Logistic Coefficients

#### Logistic regression re-fitted from 6 to 36 months (interval = 2m)

library(dplyr)

library(broom)

library(ggplot2)

library(patchwork)

# Load data

data <- read.csv("train.csv")

# 存储每个时间点的模型系数

results <- list()

# Loop time points: every 2 months (6 → 36)

for (m in seq(6, 36, by = 2)) {

# 定义新的二分类结果：是否存活到 m 月

data$surv_m <- ifelse(data$Time >= m, 1, 0)

# Logistic 回归

fit <- glm(surv_m ~ LCR + TNM, data = data, family = binomial)

# 使用 broom::tidy 提取 OR/CI/系数

coef_df <- tidy(fit, conf.int = TRUE, exponentiate = FALSE) %>%

filter(term %in% c("LCR", "TNM")) %>%

mutate(month = m)

results[[as.character(m)]] <- coef_df

}

# 合并所有时间点的系数

coef_trend <- bind_rows(results)

#### Plot 1 — Original logistic coefficients

p1 <- ggplot(coef_trend, aes(x = month, y = estimate,

color = term, group = term)) +

geom_line(size = 1) +

geom_point(size = 2) +

geom_ribbon(aes(ymin = conf.low, ymax = conf.high, fill = term),

alpha = 0.15, color = NA) +

theme_classic(base_size = 14) +

labs(x = "Time (months)",

y = "Coefficient (log-odds)",

title = "Original Coefficients Across Time") +

scale_color_manual(values = c("LCR" = "#1b9e77",

"TNM" = "#d95f02")) +

scale_fill_manual(values = c("LCR" = "#1b9e77",

"TNM" = "#d95f02"))

#### Plot 2 — Absolute coefficients

coef_trend_abs <- coef_trend %>%

mutate(estimate = abs(estimate),

conf.low = abs(conf.low),

conf.high = abs(conf.high))

p2 <- ggplot(coef_trend_abs, aes(x = month, y = estimate,

color = term, group = term)) +

geom_line(size = 1) +

geom_point(size = 2) +

geom_ribbon(aes(ymin = conf.low, ymax = conf.high, fill = term),

alpha = 0.15, color = NA) +

theme_classic(base_size = 14) +

labs(x = "Time (months)",

y = "Absolute coefficient",

title = "Absolute Coefficients Across Time") +

scale_color_manual(values = c("LCR" = "#1b9e77",

"TNM" = "#d95f02")) +

scale_fill_manual(values = c("LCR" = "#1b9e77",

"TNM" = "#d95f02"))

#### Combine plots side-by-side

final_plot <- p1 | p2

print(final_plot)

#### Save final high-resolution image (PNG)

ggsave("coef_trends_side_by_side.png",

final_plot, width = 18, height = 6, dpi = 300)
